# Antiviral Activity of Diltiazem HCl Against Pseudorabies Virus Infection In Vitro

**DOI:** 10.3390/vetsci12090864

**Published:** 2025-09-05

**Authors:** Mengting Zuo, Decai Xiang, Zhen-Xing Zhang, Xi Yang, Yuqing Duan, Juan Li, Bangquan Zeng, Lu Dong, Guoquan Wu, Yi Zhou, Lei Tan, Bofang Duan

**Affiliations:** 1 Hunan Provincial Key Laboratory of the TCM Agricultural Biogenomics, Changsha Medical University, Changsha 410219, China; zuomengting@aliyun.com; 2 Yunnan Tropical and Subtropical Animal Virus Diseases Laboratory, Yunnan Animal Science and Veterinary Institute, Kunming 650224, China; askalm@163.com (D.X.); zhenxing978@163.com (Z.-X.Z.); wuguoquan1982@163.com (G.W.); 3 College of Animal Science and Technology, Yangtze University, Jingzhou 434025, China; yangxi20030204@163.com (X.Y.); dyq202507@163.com (Y.D.); 4 Yunnan Sino-Science Gene Technology Co., Ltd., Kunming 650501, China; ynndlj@126.com; 5 Central for Animal Disease Control and Prevention of Yunnan Province, Kunming 650201, China; quanbangzeng123@163.com (B.Z.); bbaoke95@163.com (L.D.)

**Keywords:** pseudorabies virus, diltiazem HCl, antiviral activity, viral replication phase, RNA-seq

## Abstract

Pseudorabies virus (PRV) is a highly pathogenic agent that adversely impacts swine populations, leading to considerable economic losses within the Chinese pig industry. Furthermore, the potential for PRV to transmit across species from pigs to other hosts has attracted significant attention. In light of this, it is essential to identify efficacious antiviral agents for the treatment of PRV infection. In this study, we investigated the antiviral properties of Diltiazem HCl (DTZ) against PRV infection in susceptible cell lines. Our results demonstrated that DTZ significantly inhibited PRV infection in both PK15 and Vero cells. Moreover, this chemical compound exhibited antiviral activity against both variant and classical strains of PRV, as well as herpes simplex virus type 1 (HSV-1). Time-of-addition assays showed that DTZ exerted its inhibitory effect through specific interference with the virus replication process. Subsequent transcriptomic analysis via RNA sequencing indicated that the calcium signaling pathway might be involved in the antiviral properties of DTZ against PRV infection. Specifically, treatment with EGTA or calcium ion (Ca^2+^)-free medium inhibited PRV infection; this inhibitory effect was substantially mitigated upon the reintroduction of CaCl_2_. In summary, DTZ effectively suppressed PRV infection in vitro, demonstrating its potential as an antiviral agent against PRV infections.

## Simple Summary

Pseudorabies virus (PRV) infection causes substantial economic losses in the global swine industry. Moreover, PRV infections have also been documented in diverse mammalian species, including humans. In this study, we found that Diltiazem hydrochloride (DTZ) significantly inhibited PRV infection through specific interference with viral replication. Further experiments revealed that DTZ-mediated modulation of the calcium signaling pathway contributed to PRV infection. Overall, DTZ may be considered a potential therapeutic agent for the management of PRV infections.

## 1. Introduction

Pseudorabies virus (PRV), the causative agent of pseudorabies (PR), is a double-stranded linear DNA virus classified within the *Alphaherpesvirinae* subfamily of the *Orthoherpesviridae* family, alongside Herpes simplex virus type 1 (HSV-1) [1]. Swine acts as the only natural reservoir for PRV, with infecting pigs exhibiting reproductive complications in sows, neurological disorders in piglets, and severe respiratory or neurological symptoms in older pigs [2,3]. Beyond swine, PRV can infect various mammals such as ruminants, dogs, and cats, typically causing fatal outcomes for these non-natural hosts [4]. Furthermore, researchers isolated a live PRV strain from the cerebrospinal fluid sample of an infected patient in 2019, highlighting its potential zoonotic risks [5,6]. These findings underscore the necessity for developing effective treatment against PRV infections.

PRV has demonstrated widespread prevalence within swine populations and other susceptible species across diverse geographic regions, encompassing Asia (most notably China, Thailand, Japan, and Korea [2,7,8,9], Europe (with significant incidences reported in Greece, Italy, Croatia, and Spain) [10,11,12,13], and South America (Argentina and Brazil) [14,15]. The global genetic profiling of PRV strains distinguishes two different genotypes (I and II) [16]. Notably, PRV strains in the genotype I are mainly prevalent in Europe [2], while genotype II, comprising variant and classical subcategories, accounts for most PRV strains in China [6]. Since 2011, variant PRV strains have become the predominant subgenotype across most regions of China, which exhibited more heightened pathogenicity than classical strains [2].

Calcium ions (Ca^2+^) and calcium signaling pathways regulate key biological processes in mammalian cells, including cell proliferation, apoptosis, and neural signal transmission [17]. Emerging evidence suggests that these pathways also influence the life cycles of multiple viruses, such as severe acute respiratory syndrome coronavirus [18], HSV-1 [19], and Zika virus [20]. Mammalian calcium channels comprise four principal classes: voltage-dependent calcium channels (VDCCs), store-operated Ca^2+^ channels, transient receptor potential channels, and receptor-operated channels, respectively [21]. Among these, VDCCs can be further subdivided into three families: L-type calcium channels, P/Q-type, N-type, R-type calcium channels, and T-type calcium channels [22]. Notably, L-type channels participate in viral absorption, entry, and replication phases for several pathogens [23,24,25]. Specifically, Diltiazem hydrochloride (DTZ) is a Food and Drug Administration (FDA)-approved medication that functions as an L-type calcium channel blocker, this chemical agent has been shown to inhibit multiple swine viruses, including Porcine reproductive and respiratory syndrome virus [26] and Porcine deltacoronavirus [27]. However, the effects of Ca^2+^ and L-type calcium channels on PRV infection require further investigations.

This study examined the antiviral properties of DTZ against PRV infection in vitro. Our findings showed that DTZ treatment markedly suppressed PRV infection by interfering with viral replication. RNA sequencing analysis demonstrated that the calcium signaling pathway mediated DTZ’s antiviral activity against PRV. Collectively, our findings suggested that DTZ could be a viable antiviral agent for PRV infection.

## 2. Materials and Methods

### 2.1. Cells, Viruses, Chemicals, and Antibodies

Vero cells (ATC, CCL-81) and PK-15 cells (ATCC, CCL-33) were cultured in DMEM supplemented with 10% FBS and 1% penicillin-streptomycin at 37 °C under 5% CO_2_ [28,29]. Two variant PRV (PRV-HuN-LD and PRV-YuN-KM) strains and a classical PRV (PRV-HuN-XT) strain were isolated and preserved in our laboratory, which were propagated and titrated in Vero cells, respectively. The HSV-1 strain was generously provided by Prof. Chunfu Zheng (University of Calgary, Calgary, Canada).

**Figure 1:**
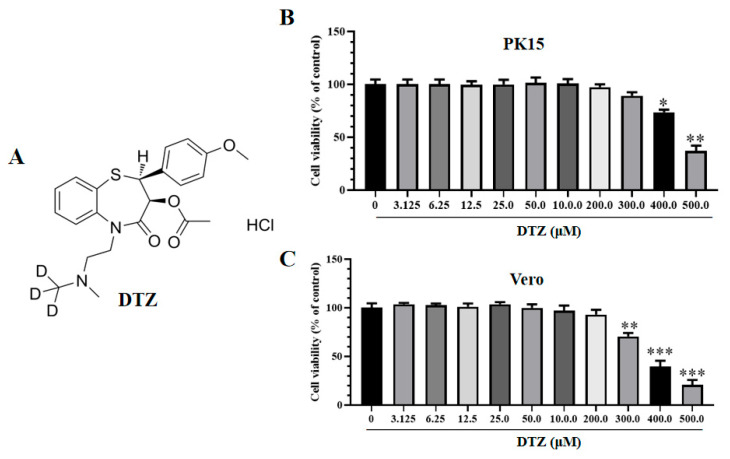
The molecular structure of DTZ and its cytotoxic effects on PK15 and Vero cells. (**A**) The molecular structure of DTZ. (**B**,**C**) Cells were exposed to varying concentrations of DTZ (ranging from 0 to 500 μM) for 48 h, after which cell viability was evaluated using the CCK-8 assay (%). * *p* < 0.05, ** *p* < 0.01, and *** *p* < 0.001 compared to the negative control.

The PRV gC protein antibody was acquired from Guangzhou Qianxun Biotechnology Co., Ltd. (Guangzhou, China). DTZ (illustrated in Figure 1A) was obtained from MedChemExpress (Shanghai, China) and dissolved in DPEC-treated water to achieve a working concentration of 200 mM. The extracellular Ca^2+^ chelator EGTA was purchased from BioFroxx and prepared in DPEC-treated water at 0.5 M.

### 2.2. Cytotoxicity Assay

Cells were seeded in a 96-well plate until reaching approximately 50% confluency, then incubated with DTZ (0, 3.125, 6.25, 12.5, 25.0, 50.0, 100.0, 200.0, and 300.0 μM) or EGTA (0, 1.0, 1.5, and 2.0 mM) at specified concentrations for 48 h. Cell viability was subsequently measured in each group using the CCK-8 method following the manufacturer’s protocol [28].

### 2.3. Nucleic Isolation and Quantitative Real-Time PCR Assessment

Genomic DNA was recovered from PRV-infected cells employing a DNA extraction kit (Takara, Dalian, China). The quantification of viral copies was performed via absolute quantification PCR (qPCR) with the recombinant plasmid pcUmT-PRV-gB and gB-specific primers, following the established protocols [28].

For RNA analysis, we isolated total RNA genome using the SteadyPure Universal RNA Extraction Kit (Accurate Biotechnology, Changsha, China), and synthesized cDNA from 1.0 μg RNA with PrimerScript RT Master Mix (Accurate Biotechnology). Relative gene expression was assessed by RT-qPCR [29], normalized to the GAPDH gene. The primer sequences were listed in Table 1.

### 2.4. Virus Titration

Vero cells were seeded in 96-well plates to achieve approximately 40% monolayer coverage. The cells were infected with ten-fold serial dilutions of viral supernatant. Cytopathic effects (CPE) were assessed and recorded at 72 h post-infection (hpi), with 50% tissue culture infectious dose (TCID_50_) values calculated using the Reed-Muench method.

### 2.5. Indirect Immunofluorescent Assay (IFA)

PRV-infected cells were fixed with 4% paraformaldehyde for 15 min and permeabilized using 0.1% Triton X-100 in PBS for 12 min at room temperature. Following a 2 h incubation at room temperature in PBS containing 3% BSA for blocking, the cells were incubated overnight at 4 °C with a monoclonal anti-PRV gC mouse antibody (1:2000). Following five PBS washes, the cells were exposed to an FITC-conjugated secondary antibody for one hour at room temperature under dark conditions. After five additional PBS washes, specific fluorescence signals were visualized by fluorescence microscopy.

### 2.6. Antiviral Activity Analysis

PK15 and Vero cells were grown in 12-well plates until reaching 90% confluency. The cells then received a 2 h pretreatment with DTZ at concentrations of 0, 50, 100, and 200 μM. Subsequently, the cells were incubated with the PRV-HuN-LD strain (MOI = 0.1) for 2 h. After PBS washing, the cells were maintained in DMEM supplemented with 10% PBS and the respective DTZ concentrations. RT-qPCR and IFA analyses were performed on harvested cells at 24 hpi, while supernatants were collected for TCID_50_-based viral titer determination.

To further assess the antiviral properties of DTZ against additional variant and classical PRV strains, as well as an HSV-1 strain, we pretreated Vero cells for 2 h with DMEM containing 10% FBS and either 100 μM or 200 μM DTZ before infecting them with one of two additional PRV strains or with HSV-1. Following infection, cells were washed with PBS and cultured in DMEM containing 10% FBS and the respective DTZ concentrations for 24 h. Subsequently, we collected the supernatants for viral titer quantification and harvested the cells to measure viral gene mRNA expression levels.

### 2.7. Inhibitory Action Assay

#### 2.7.1. Inactivation Assay

An equal volume of PRV-HuN-LD viral solution (10^5^ TCID_50_) was mixed with DMEM containing 200 μM DTZ and incubated at 37 °C for 2 h. The mixture was then added to Vero cells cultured in a 6-well plate at approximately 80% confluence and incubated for 2 h at 37 °C. After washing with PBS, the supernatants were replaced with DMEM supplemented with 2% FBS. Viral titers were assessed in supernatants collected at 24 hpi.

#### 2.7.2. Pre-Treatment Assay

Vero cells were grown in a 6-well plate until reaching 80% confluence. The cells were treated with DMEM containing 10% FBS, with or without 100 μM DTZ at room temperature for 2 h. Following PBS washing, the cells were infected with 10^5^ TCID_50_ of PRV-HuN-LD and incubated at 37 °C in a 5% CO_2_ incubator for 2 h. After a second PBS wash, the medium was replaced with DMEM containing 2% FBS. At 24 hpi, viral titers were measured in supernatants.

#### 2.7.3. Viral Attachment Assay

Vero cells were cultured in a 6-well plate and reached approximately 80% confluence. After incubation with DMEM containing 10% FBS, with or with 100 μM DTZ, at 4 °C for 2 h, the cells were exposed to PRV-HuN-LD at varying MOIs (0.1, 1.0, and 10.0) under the same conditions to facilitate viral attachment. Following washes with pre-cooled PBS, total cellular DNA was extracted employing a DNA/RNA extraction kit (Takara, Dalian, China). Viral copy numbers were quantified by qPCR.

#### 2.7.4. Viral Entry Assay

Vero cells were cultured in a 6-well plate until they reached approximately 80% confluence. The cells were incubated with DMEM containing PRV-HuN-LD at MOI of 0.1, 1.0, and 10.0 for 2 h at 4 °C to allow viral attachment. After PBS washes, cells were treated with DMEM supplementary with 10% FBS, with or without 100 μM DTZ, for 2 h at 37 °C in a 5% CO_2_ incubator to enable viral entry. After additional PBS washes, total cellular DNA was extracted using a DNA/RNA extraction kit (Takara, Dalian, China). Viral copy numbers were quantified by qPCR.

#### 2.7.5. Virus Replication Assay

Vero cells were cultured in 6-well plates until they reached approximately 80% confluence. The cells were then incubated with DMEM containing 10^5^ TCID_50_ of PRV-HuN-LD at 37 °C in a 5% CO_2_ incubator for 2 h to facilitate viral entry. Following PBS washing, the cells were maintained in DMEM supplemented with 10% FBS, with or without DTZ (100 μM), under the same incubation conditions. At 24 hpi, viral titers in the collected supernatants were assessed using the TCID_50_ assay.

#### 2.7.6. Virus Release Assay

Vero cells were cultured in a 6-well plate until they reached nearly 80% confluence. The cells were then incubated with DMEM containing 10^5^ TCID_50_ of PRV-HuN-LD at 37 °C under 5% CO_2_ incubator for 2 h to allow viral entry. After washing with PBS, the cells were maintained in DMEM supplemented with 2% FBS at 37 °C under the same incubation conditions. At 20 hpi, the medium was replaced with DMEM containing 10% FBS, either with or without DTZ (100 μM). Viral titers were assessed by TCID_50_ assay after collecting supernatants at 24 hpi and 26 hpi.

### 2.8. Assessment of the Effect of Intracellular Ca^2+^ on PRV Infection

To assess the effect of intracellular Ca^2+^ on PRV infection in vitro, we cultured Vero and PK15 cells in DMEM with or without Ca^2+^ in 6-well plates until they reached nearly 80% confluence. The cells were then infected with 10^5^ TCID_50_ of PRV-HuN-LD at 37 °C in a 5% CO_2_ incubator for 2 h to facilitate viral entry. After washing with PBS, the cells were maintained in DMEM containing either 2 mM CaCl_2_ or no additional Ca^2+^. At 24 hpi, the supernatants and cells were harvested to assess viral titers by TCID_50_ assay and viral copies numbers by qPCR method, respectively.

In a separate experiment, Vero cells were pretreated with EGTA with or without CaCl_2_ for 2 h, then infected with 10^5^ TCID_50_ of PRV-HuN-LD at 37 °C under a 5% CO_2_ incubator for 2 h to allow viral entry. Following PBS washes, the cells were maintained in their respective treatment media. Viral titers and copy numbers were similarly assessed at 24 hpi using TCID_50_ and qPCR, respectively.

### 2.9. Experiment Design, cDNA Library Construction, and Sequence Date Analysis

PK15 cells were grown in 6-well plates until reaching nearly 80% confluence. The cells were then challenged with PRV-HuN-LD (MOI = 0.1) for 2 h. After a PBS rinse, the cells were maintained in DMEM containing 10% FBS, with or without DTZ (100 μM), at 37 °C under 5% CO_2_. At 24 hpi, cells were washed with chilled PBS, total RNA was extracted using the SteadyPure Universal RNA Extraction Kit (Accurate Biotechnology, Changsha, China). RNA samples were sent to Wuhan Jinkairui Biotechnology Co., Ltd. (Wuhan, China) for sequencing, as previously described [29].

### 2.10. Statistical Analysis

Each experiment was performed in triplicate with independent replicates. The results were expressed as mean ± standard deviation. Data derived from different experimental groups were analyzed using two-tailed Student’s *t*-tests in GraphPad Prism Version 8.0 (GraphPad Software, La Jolla, CA, USA). Statistical significance was defined as * *p* < 0.05, ** *p* < 0.01, and *** *p* < 0.001, while *p* > 0.05 was considered not significant.

## 3. Results

### 3.1. Cytotoxicity of DTZ on Different Cell Lines

The cytotoxicity of DTZ was evaluated in PK15 and Vero cells using a CCK-8 kit. As shown in Figure 1B,C, both PK15 and Vero cells treated with 200 μM of DTZ exhibited no significant cytotoxic effects compared to the untreated group. The 50% cytotoxicity concentration (CC_50_) values for DTZ were 478.26 ± 12.27 μM in PK15 cells and 341.77 ± 10.80 μM in Vero cells.

### 3.2. DTZ Significantly Inhibits PRV-HuN-LD Infection in Both Vero and PK15 Cell Lines

**Figure 2:**
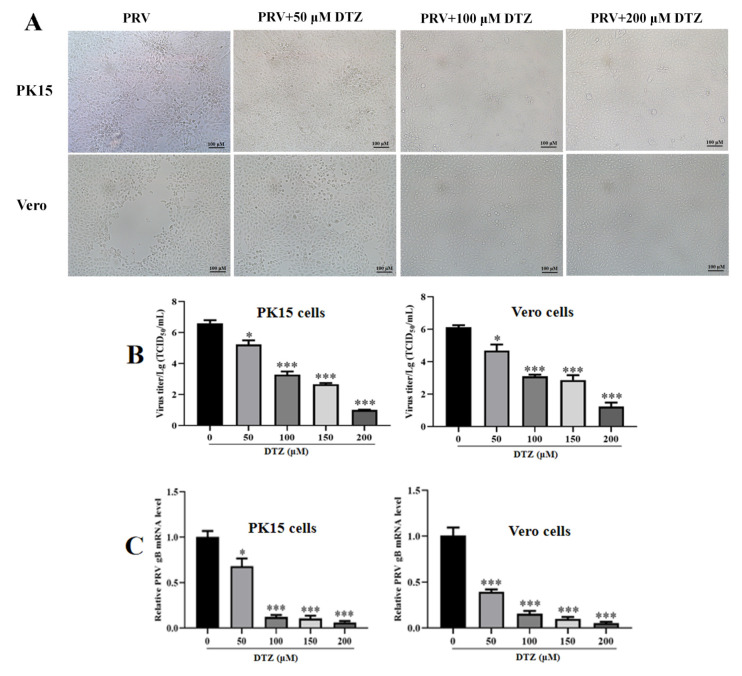

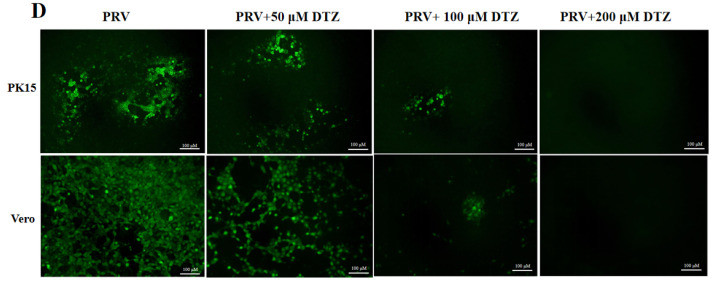
Antiviral activity of DTZ against PRV infection in vitro. (**A**) PK15 and Vero cell were pretreated with DTZ (50.0, 100.0, and 200.0 μM) for 2 h, and exposed to PRV (MOI = 0.1). Following a 24-hpi period, cell morphology was observed. (**B**–**D**) PK15 and Vero cell lines were pretreated with DTZ (50.0, 100.0, 150.0, and 200.0 μM) for 2 h, and exposed to PRV (MOI = 0.1). Following 24 h of incubation, paired cell lysates and supernatants were collected for TCID_50_ titration (**B**), RT-qPCR analysis (**C**), and IFA (**D**). * *p* < 0.05 and *** *p* < 0.001 compared to the negative control.

The antiviral efficacy of DTZ against PRV-HuN-LD strain was assessed in vitro. DTZ treatment substantially mitigated PRV-induced cytopathic effects in both PK15 and Vero cells (Figure 2A). Administration of DTZ at concentrations between 50 and 200 μM significantly decreased PRV-gB expression and viral titers in PK15 and Vero cells (Figure 2B,C). IFA analysis revealed intensive PRV-specific fluorescence in untreated controls, whereas DTZ treatment dose-dependently attenuated these signals (Figure 2D). The 50% maximal inhibitory concentration (IC_50_) values for DTZ were 39.28 ± 1.33 μM in PK15 cells and 25.53 ± 2.59 μM in Vero cells, yielding selectivity index (SI) values of 12.17 and 13.38, respectively (Table 2).

### 3.3. DTZ Inhibits PRV-HuN-LD Infection by Targeting Viral Replication Stage

**Figure 3:**
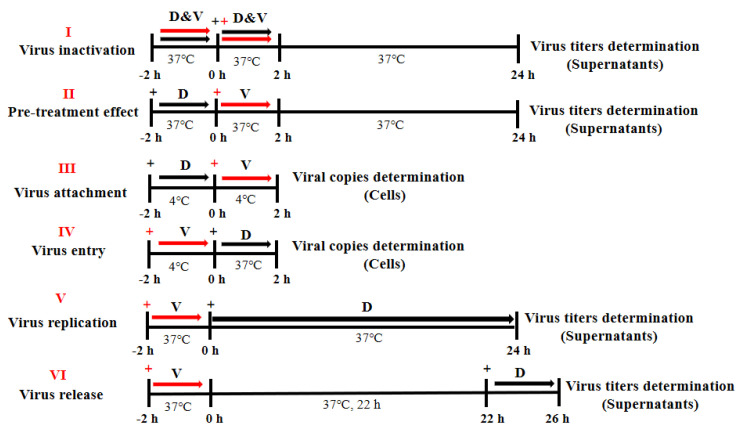

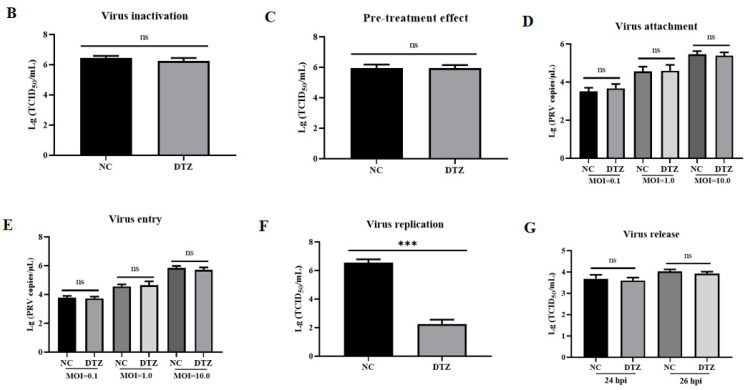
Effects of DTZ on distinct stages of the PRV replication cycle in Vero cells. (**A**) A schematic representation of virus inactivation (I), pre-treatment (II), virus attachment (III), virus entry (IV), virus replication (V), and virus release (VI). At the corresponding time points, cells or supernatants were collected for viral titer (**B**,**C**,**F**,**G**) and viral copy determination (**D**,**E**). *** *p* < 0.001 and ns *p* > 0.05 compared to the negative control.

The antiviral efficacy of DTZ against PRV infection was evaluated by examining its effect at various treatment time points in Vero cells (Figure 3A). The results demonstrated that viral titers and copy numbers remained unchanged across experimental models testing which included virus inactivation (Figure 3B), pre-treatment (Figure 3C), attachment (Figure 3D), entry (Figure 3E), as well as release (Figure 3G). However, DTZ treatment significantly reduced progeny viral titers specifically during the replication phase of PRV infection (Figure 3F).

### 3.4. DTZ Exhibits Antiviral Efficacy Against PRV and HSV-1

**Figure 4:**
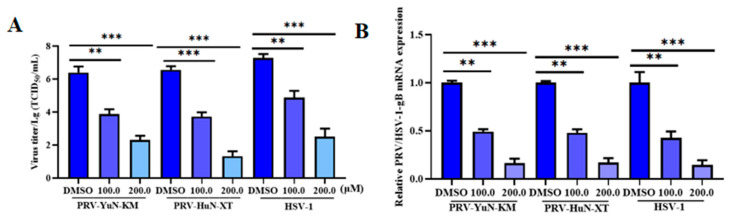
DTZ impeded the infections by multiple alphaherpesvirus strains in vitro. After a 2 h pretreatment with 100 or 200 μM, cells were challenged with distinct virus strains. Paired supernatants and cell lysates were collected for viral titer quantification (**A**) and RT-qPCR analysis (**B**). ** *p* < 0.01 and *** *p* < 0.001 compared to the negative control.

To assess the antiviral property of DTZ against alphaherpesvirus infection in vitro, other strains, including two PRV strains and an HSV-1 strain, were utilized to further investigate the antiviral activity of DTZ in Vero cells. As shown in Figure 4, DTZ treatment significantly reduced viral titers for all three strains in a dose-dependent manner (Figure 4A). Similarly, viral gene mRNA transcription levels for both PRV and HSV-1 were substantially lower in DTZ-treated groups compared to controls (Figure 4B).

### 3.5. Transcription Analysis of PK15 Cells Infected with PRV Co-Treated with DTZ

**Figure 5:**
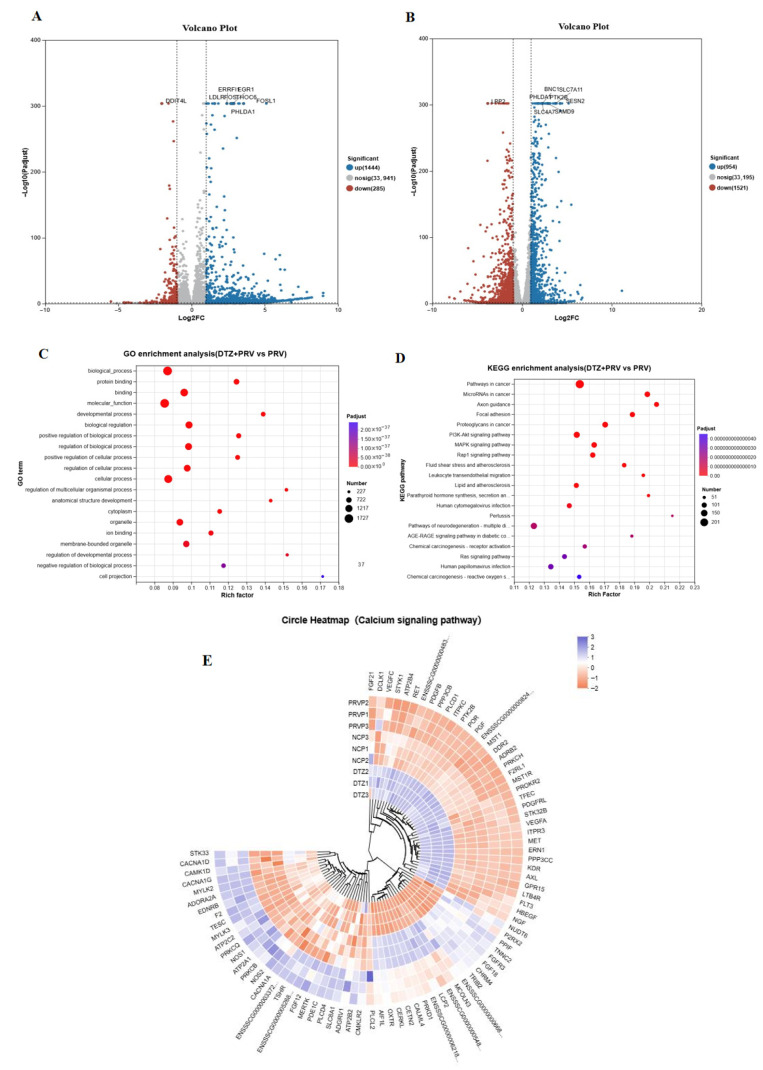
Transcriptomic insights into the mechanism of DTZ-mediated antiviral activity. (**A**,**B**) Volcano plots depict the DEG profiles: mock vs. PRV-infected PK15 cells (**A**) and PRV-infected vs. PRV+DTZ-treated PK15 cells (**B**), with red and green dots denoting significantly down- and up-regulated genes, respectively. (**C**,**D**) Functional profiling of the DEGs between PRV-infected and DTZ+PRV-infected PK15 cells by Gene Ontology (**C**) and KEGG (**D**) enrichment analyses. (**E**) Circle heatmap analysis of the DEGs associated with the calcium signaling pathway. Note: the designations PRVP1-3, NCP1-3, and DTZ1-3 correspond to the PRV-infected group, the negative control group, and the DTZ-treated PRV-infected group, respectively.

To examine the cellular pathways involved in the antiviral effects of DTZ against PRV infection, we conducted RNA sequencing analyses on PRV-infected PK15 cells treated with or without DTZ. The analysis revealed 1729 differentially expressed genes (DEGs) between PRV-infected and mock-infected groups, including 1444 up-regulated DEGs and 285 down-regulated DEGs. In contrast, DTZ-treated PRV-infected PK15 cells showed 2475 DEGs, comprising 954 up-regulated DEGs and 1521 down-regulated DEGs (Figure 5A,B).

The Kyoto Encyclopedia of Genes and Genomes (KEGG) analysis indicated significant enrichment of DEGs between PRV-infected and DTZ-treated groups in cancer-related pathway, axon guidance, focal adhesion, proteoglycans in cancer, the PI3K-Akt signaling pathway and the MAPK signaling pathway (Figure 5C,D), all of which are modulated by calcium signaling [19,20]. A cluster analysis of DEGs in the calcium signaling pathway demonstrated that PRV infection led to an upregulation of mRNA expression levels of several calcium signaling-associated genes, including CACNA1D, CACNA1A, CAMK1D, CACNA1G, NOS1, and NOS2 (Figure 5E). Conversely, key genes involved in calcium signaling, such as STYK1, FGF21, ATP2B4, PRKCH, MST1, and DDR2, were downregulated following PRV infection (Figure 5E). Notably, these alterations were reversed upon treatment with DTZ.

### 3.6. Validation of Transcriptional Levels of DEGs Using RT-qPCR

**Figure 6:**
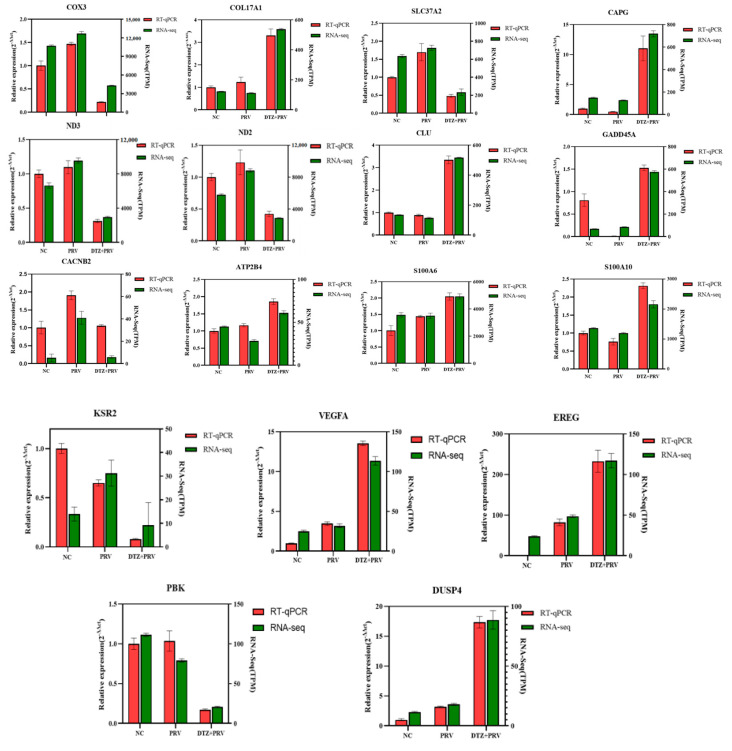
Validation of the mRNA expression levels of representative DEGs identified in the RNA-Seq data though RT-qPCR.

We next assessed the accuracy and reliability of RNA-Seq results by measuring transcriptional levels of pathway-associated genes via RT-qPCR. Key targets including antioxidative stress genes (COX3, ND2, and ND3), epithelial–mesenchymal transition signaling markers (EMILIN2, CLDN4, CAPG, COL17A1, and EMP1), calcium signaling pathway (CACNB2, ATP2B4, S100A6, and S100A10), and MAPK pathway regulators (KSR2, VEGFA, EREG, GADD45A, and DUSP4) were randomly selected for further analysis. As illustrated in Figure 6, the RT-qPCR data demonstrated a high level of concordance with the RNA-Seq results. Although the magnitude of downregulation or upregulation differed for several selected genes, such as S100A6 and GADD45A, these results collectively indicated that the RNA-Seq data possessed considered accuracy and reproducibility.

### 3.7. Ca^2+^ Uptake Is Essential for PRV Infection In Vitro

**Figure 7:**
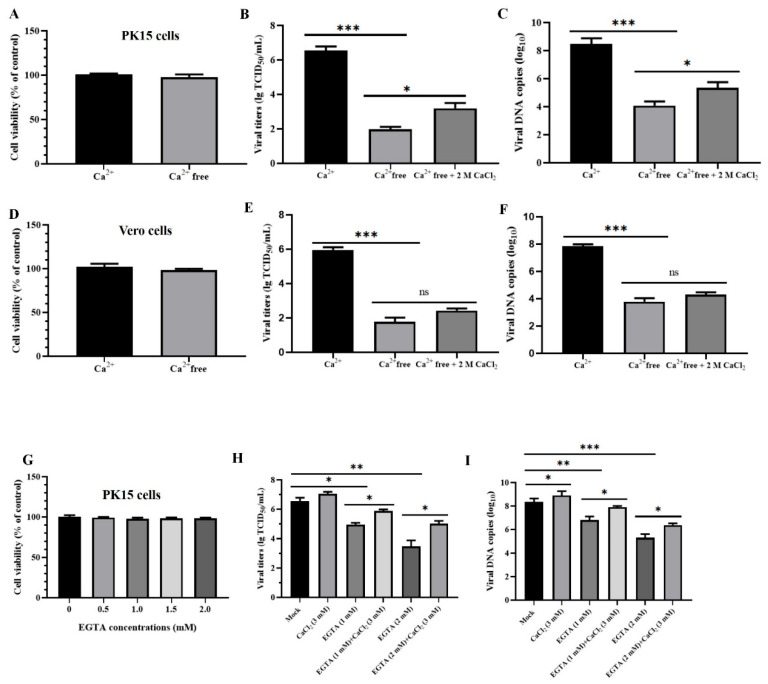
Ca^2+^ uptake is required for PRV infection in vitro. (**A**,**D**) Cell variability of PK15 and Vero cells cultured in DMEM with or without Ca^2+^. (**B**,**C**,**E**,**F**) Cells were cultured in DMEM supplemented with or without Ca^2+^, then infected with PRV at an MOI of 0.1. At 24 hpi, supernatants and cells were harvested for viral titer (**B,E**) and viral copy number analysis (**E**,**F**). (**G**) PK15 cells were treated with varying concentrations of EGTA (0, 0.5, 1.0, 1.5, and 2.0 mM) for 48 h, after which cell viability was evaluated using the CCK-8 assay (%). (**H**,**I**) PK15 cells were treated with EGTA or/and CaCl_2_ for 2 h, then challenged with PRV (MOI = 0.1). At 24 hpi, paired supernatants and cell lysates were collected for viral titer (**H**) and viral copy number analysis (**I**). * *p* < 0.05, ** *p* < 0.01, *** *p* < 0.001 and ns *p* > 0.05 compared to the negative control.

The aforementioned results demonstrated that the calcium signaling pathway mediated DTZ’s antiviral activity against PRV infection. As a calcium channel blocker, DTZ selectively inhibits calcium ion influx through L-type voltage-gated calcium channels in cardiac and vascular smooth muscle cells [30]. We therefore investigated how Ca^2+^ uptake influences PRV infection by comparing viral replication in PK15 and Vero cells cultured in normal versus Ca^2+^-free DMEM medium at different time points. As illustrated in Figure 7, Ca^2+^ deletion caused no significant cytotoxicity in either PK15 or Vero cells (Figure 7A,D), yet markedly decreased viral copies and titers; however, the introduction of Ca^2+^ substantially restored this inhibitory effect (Figure 7B,C,E,F). EGTA treatment similarly reduced viral loads in PK15 cell, with CaCl_2_ incubation reversing this suppression (Figure 7G–I).

## 4. Discussion

PRV has become a significant infectious disease that severely impacts Chinese pig industry. Its cross-species transmission capability from pigs to other mammals has raised significant public health concerns [16]. Although eradication efforts involving vaccination programs and improved biosecurity measures persist, field PRV strains maintain high prevalence in specific regions or provinces in China [31,32,33]. Developing antiviral agents could provide an additional approach for PRV control. Nevertheless, no licensed agents currently exist for treating PRV infections.

DTZ, an FDA-approved pharmacological agent, functions as an L-type voltage-gated calcium channel blocker [34]. It has been extensively employed for calcium signaling-related disorders including hypertension [35], angina pectoris [36], and arrhythmias [37]. In recent years, its potential antiviral applications of DTZ have gained attention due to calcium signaling’s involvement in viral pathogenesis [21,37,38]. Our study demonstrated DTZ’s significant inhibition of PRV infection in PK15 and Vero cells, with selectivity indices of 12.17 and 13.38, respectively. This compound also demonstrated broad-spectrum activity against classical and variant PRV strains, as well as HSV-1, indicating potential efficacy against alphaherpesvirus infections. Additionally, the antiviral effects of DTZ have extended to other swine pathogens such as PRRSV and PDCoV, further investigations will determine its antiviral potential in porcine models [26,27].

The viral life cycle encompasses attachment, entry, replication, and release phases [1]. DTZ inhibits virus replication through distinct mechanisms across different viruses. For example, DTZ blocked PRRSV infection during internalization and post-entry stages [26], whereas it reduced PDCoV infection by interfering with the replication stage [27]. Though both PDCoV and SARS-CoV-2 belong to the *Coronaviridae* family, DTZ suppressed SARS-COV-2 infection by impairing viral binding and internalization [39]. In the present study, DTZ inhibited PRV infection exclusively during replication, without affecting viral binding, entry, or release stages. Verapamil, another L-type Ca^2+^ calcium channel blocker, prevented bovine herpesvirus 1 (BHV-1) replication during post-entry stage [40]. Conversely, similar pharmacological agents (nifedipine and nitrendipine) exhibited no antiviral activity against HSV-2 [41]. Collectively, these findings suggest that the involvement of L-type calcium channels in viral infections is heterogeneous, though the viruses being classified within the same family.

RNA-Seq technology has become a standard tool for analyzing comprehensive transcriptional changes occurring in the host cells [42,43,44]. This study employed RNA-Seq technology to characterize how DTZ exerted antiviral effects against PRV infection in PK15 cells. KEGG pathway analysis revealed DTZ mediated modulation of several signaling pathways during PRV infection, including MAPK, PI3K-Akt, and cGAMP-PKG signaling pathways. Notably, both MAPK and PI3K-Akt pathways are implicated in viral pathogenesis [45,46], and have been implicated in the antiviral properties of other compounds against herpesvirus infections, such as Myricetin (against PRV) and Paeonol (against BHV-1) [47,48]. Since these pathways are modulated by calcium, and DTZ blocks L-type calcium channels, we specifically analyzed the transcriptional alterations of calcium-related DEGs. PRV infection up-regulated CACNA1D, CACNA1A, CAMK1D, CACNA1G, NOS1, and NOS2 expression, consistent with calcium pathway activation. However, DTZ treatment suppressed this response. Subsequent experiments demonstrated that extracellular Ca^2+^ deletion or chelation markedly reduced PRV replication, mirroring reported effects on PRRSV [26] and PDCoV [27].

It should be noted that this study has not fully elucidated the antiviral mechanisms of DTZ against PRV infection. Firstly, DTZ treatment significantly impedes viral replication, whether it directly interacts with viral proteins remains uncertain. Secondly, DTZ primarily targets the calcium voltage-gated channel subunit alpha 1C protein, which participates in PRRSV [26], PDCoV [27], and SARS-COV-2 [18] infections; but its role in PRV infection requires further investigation. Thirdly, in vivo antiviral efficacy must be evaluated through animal studies. Additionally, the translational potential and safety profile of DTZ for the treatment of PRV in swine warrant thorough investigation.

In conclusion, our findings revealed that DTZ exerted a potent anti-PRV effect in vitro by arresting viral replication. Moreover, DTZ-mediated modulation of the calcium signaling pathway contributed to PRV infection. These findings indicated that DTZ could be a viable therapeutic option for the management of PRV.

## Figures and Tables

**Table 1:** Oligonucleotide primers employed in this investigation.

Gene	Sequence (3′–5′)	GenBank Accession Number
q-PRV-*gB*-F	GTCCGTGAAGCGGTTCGTGAT	OP879616
q-PRV-*gB*-R	CTCCATCATGAAGTGCGACGT	
q-HSV-*gB*-F	GGACATCAAGGCGGAGAACA	MH999849
q-HSV-*gB*-R	TTCTCCTTGAAGACCACCGC	
q-*GAPDH*-F	ACCACAGTCCATGCCATCAC	OZ289217
q-*GAPDH*-R	TCCACCACCCTGTTGCTGTA	
*EMILIN2*-qPCR-F	CGCCAGGAACAAGAACTGGTG	NC_010448
*EMILIN2*-qPCR-R	GCACAGTTGTACTGAGCCTGA	
*ND2*-qPCR-F	AATCCACAGCTCAGCAACCA	MK688993
*ND2*-qPCR-R	TTAGGCTTGTGATGACGGGT	
*ND3*-qPCR-F	AACCCTAGCCTCCCTACTCG	MK688993
*ND3*-qPCR-R	GAGGCGTGCTGATCCTATGG	
*SLC37A2*-qPCR-F	TGTGGTCAAGAGTCGTCTGC	XM_021063086
*SLC37A2*-qPCR-R	ATGCCGATAGCATAGGCCAC	
*COX3*-qPCR-F	ACCACTTACCGGAGCCCTAT	MK688993
*COX3*-qPCR-R	ATGTGTGGTGGCCTTGGAAA	
*COL17A1*-qPCR-F	TCCTTACCACCAAAAGGGGG	XM_071611703
*COL17A1*-qPCR-R	AACTGGAGGTGGAGGCATTG	
*CLDN4*-qPCR-F	TGGATGATGAGAGCGCCAAG	NM_001161637
*CLDN4*-qPCR-R	GGGATTGTAGAAGTCGCGGA	
*CAPG*-qPCR-F	GACTCAGAGCTGCTAGCCTT	AK238228
*GAPG*-qPCR-R	TGCTGTTTCCAGATCTCCTCC	
*EMP1*-qPCR-F *EMP1*-qPCR-R	CATGCTGTTCGTTTGCACCA ACTTGAGGGCATCTTCACCG	AK391109
*CACNB2*-qPCR-F	GTCACCTGATGAGGAGTCTGC	XM_021064820
*CACNB2*-qPCR-R	AGTGTCAGACGAAGTGCTCC	
*ATP2B4*-qPCR-F	CGAGATTGACCACGCAGAGA	XM_021063199
*ATP2B4*-qPCR-R	GCTCCCGTCTGGAATGTGTT	
*SA100A6*-qPCR-F	ATGCCCTCTGGATCAGGCTA	AY610306
*SA100A6*-qPCR-R	GCCCCAATGGTGAGTTCCTT	
S100A10-qPCR-F	AAAAGACCCTCTGGCTGTGG	AC277996
S100A10-qPCR-R	GCCCAGCGATTAGCGAAAAG	

**Table 2:** Antiviral activity of DTZ against PRV in vitro.

Compound	Cell Lines	CC_50_ (μM)	IC_50_ (μM)	SI
DTZ	Vero	341.77 ± 10.80	25.53 ± 2.59	13.38
	PK15	478.26 ± 12.27	39.28 ± 1.33	12.17

## Data Availability

All datasets in this study can be found in the article.

## References

[1] Tan L., Wang K., Bai P., Zhang S., Zuo M., Shu X., Wang A., Yao J. (2023). Host cellular factors involved in pseudorabies virus attachment and entry: A mini review. Front. Vet. Sci..

[2] Tan L., Yao J., Yang Y., Luo W., Yuan X., Yang L., Wang A. (2021). Current status and challenge of pseudorabies virus infection in China. Virol. Sin..

[3] Chen H., Fan J., Sun X., Xie R., Song W., Zhao Y., Yang T., Cao Y., Yu S., Wei C. (2023). Characterization of pseudorabies virus associated with severe respiratory and neuronal signs in old pigs. Transbound. Emerg. Dis..

[4] Liu Q., Kuang Y., Li Y., Guo H., Zhou C., Guo S., Tan C., Wu B., Chen H., Wang X. (2022). The epidemiology and variation in pseudorabies virus: A continuing challenge to pigs and humans. Viruses.

[5] Liu Q., Wang X., Xie C., Ding S., Yang H., Guo S., Li J., Qin L., Ban F., Wang D. (2021). A novel human acute encephalitis caused by pseudorabies virus variant strain. Clin. Infect. Dis..

[6] Wei L., Hu Y., Bai L., Xiao C., Liu Z., You Y., Wang K., Huang Y., Zhu J., Weng J. (2025). Design of the inhibitors for pseudorabies virus replication by reinforcement learning from HSV-1 DNA polymerase inhibitors. ACS Omega.

[7] Panyasing Y., Kedkovid R., Kittawornrat A., Ji J., Zimmerman J., Thanawongnuwech R. (2018). Detection of Aujeszky’s disease virus DNA and antibody in swine oral fluid specimens. Transbound. Emerg. Dis..

[8] Mahmoud H.Y., Suzuki K., Tsuji T., Yokoyama M., Shimojima M., Maeda K. (2011). Pseudorabies virus infection in wild boars in Japan. J. Vet. Med. Sci..

[9] Truong Q.L., Seo T.W., Yoon B.I., Kim H.C., Han J.H., Hahn T.W. (2013). Prevalence of swine viral and bacterial pathogens in rodents and stray cats captured around pig farms in Korea. J. Vet. Med. Sci..

[10] Papageorgiou K., Stoikou A., Papadopoulos D.K., Tsapouri-Kanoula E., Giantsis I.A., Papadopoulos D., Stamelou E., Sofia M., Billinis C., Karapetsiou C. (2024). Pseudorabies Virus Prevalence in Lung Samples of Hunted Wild Boars in Northwestern Greece. Pathogens.

[11] Ferrara G., Pagnini U., Parisi A., Amoroso M.G., Fusco G., Iovane G., Montagnaro S. (2024). A pseudorabies outbreak in hunting dogs in Campania region (Italy): A case presentation and epidemiological survey. BMC Vet. Res..

[12] Konjević D., Sučec I., Turk N., Barbić L., Prpić J., Krapinec K., Bujanić M., Jemeršić L., Keros T. (2023). Epidemiology of Aujeszky disease in wild boars (*Sus scrofa* L.) in Croatia. Vet. Res. Commun..

[13] Cano-Terriza D., Martínez R., Moreno A., Pérez-Marín J.E., Jiménez-Ruiz S., Paniagua J., Borge C., García-Bocanegra I. (2019). Survey of Aujeszky’s Disease Virus in Hunting Dogs from Spain. Ecohealth.

[14] Serena M.S., Cappuccio J., Fossaroli M., Williman M.M., Dibarbora M., Brizzio R., Metz G., Aspitia C., Perez A., Carpinetti B. (2023). Characterization of new strains of Pseudorabies virus in Argentina: Detection of interspecies transmission. Open Vet. J..

[15] Kmetiuk L.B., Cassaro Villalobos E.M., do Carmo Custódio de Souza Hunold Lara M., Machado F.P., Lipinski L.C., Dos Santos A.P., de Barros Filho I.R. (2020). Serosurvey for Pseudorabies (Aujeszky’s Disease) in Free-Range Wild Boars (*Sus scrofa*) of Brazil. J. Wildl. Dis..

[16] He W., Auclert L.Z., Zhai X., Wong G., Zhang C., Zhu H., Xing G., Wang S., He W., Li K. (2019). Interspecies Transmission, Genetic Diversity, and Evolutionary Dynamics of Pseudorabies Virus. J. Infect. Dis..

[17] Bagur R., Hajnóczky G. (2017). Intracellular Ca^2+^ sensing: Its role in calcium homeostasis and signaling. Mol. Cell.

[18] Berlansky S., Sallinger M., Grabmayr H., Humer C., Bernhard A., Fahrner M., Frischauf I. (2022). Calcium signals during SARS-CoV-2 infection: Assessing the potential of emerging therapies. Cells.

[19] Cheshenko N., Del Rosario B., Woda C., Marcellino D., Satlin L.M., Herold B.C. (2003). Herpes simplex virus triggers activation of calcium-signaling pathways. J. Cell Biol..

[20] Doñate-Macián P., Jungfleisch J., Pérez-Vilaró G., Rubio-Moscardo F., Perálvarez-Marín A., Diez J., Valverde M.A. (2018). The TRPV4 channel links calcium influx to DDX3X activity and viral infectivity. Nat. Commun..

[21] Chen X., Cao R., Zhong W. (2019). Host calcium channels and pumps in viral infections. Cells.

[22] Berridge M.J., Bootman M.D., Roderick H.L. (2003). Calcium signalling: Dynamics, homeostasis and remodelling. Nat. Rev. Mol. Cell Biol..

[23] Li H., Zhang L.K., Li S.F., Zhang S.F., Wan W.W., Zhang Y.L., Xin Q.L., Dai K., Hu Y.Y., Wang Z.B. (2019). Calcium channel blockers reduce severe fever with thrombocytopenia syndrome virus (SFTSV) related fatality. Cell Res..

[24] Fujioka Y., Nishide S., Ose T., Suzuki T., Kato I., Fukuhara H., Fujioka M., Horiuchi K., Satoh A.O., Nepal P. (2018). A sialylated voltage-dependent Ca^2+^ channel binds hemagglutinin and mediates influenza A virus entry into mammalian cells. Cell Host Microbe.

[25] Lavanya M., Cuevas C.D., Thomas M., Cherry S., Ross S.R. (2013). siRNA screen for genes that affect Junín virus entry uncovers voltage-gated calcium channels as a therapeutic target. Sci. Transl. Med..

[26] Li L., Wang J., Chen L., Ren Q., Akhtar M.F., Liu W., Wang C., Cao S., Liu W., Zhao Q. (2024). Diltiazem HCl suppresses porcine reproductive and respiratory syndrome virus infection in susceptible cells and in swine. Vet. Microbiol..

[27] Bai D., Fang L., Xia S., Ke W., Wang J., Wu X., Fang P., Xiao S. (2020). Porcine deltacoronavirus (PDCoV) modulates calcium influx to favor viral replication. Virology.

[28] Xiong K., Tan L., Yi S., Wu Y., Hu Y., Wang A., Yang L. (2022). Low-concentration T-2 toxin attenuates pseudorabies virus replication in porcine kidney 15 cells. Toxins.

[29] Tan L., Zhu P., Getu Z., Yang X., Zheng S., Duan Y., Wang J., Wang J., Zhou Y., Hu Y. (2025). Antiviral activity of nitazoxanide against pseudorabies virus infection in vitro. Front. Vet. Sci..

[30] Rosales C., Brown E.J. (1992). Calcium channel blockers nifedipine and diltiazem inhibit Ca^2+^ release from intracellular stores in neutrophils. J. Biol. Chem..

[31] Zhao M., Chen J., Luo S., Zhang P., Chen J., Sun C., Ren Z., Huang Y., Zhang X., Xiang H. (2025). Epidemiological investigation, related factors, spatial-temporal cluster analysis of pseudorabies virus seroprevalence in Guangdong Province of China. Front. Vet. Sci..

[32] Sun Y., Shi M., Yang H., Zhang X., Zhang Y., Liu R., Li L., Li S., Zhou X., Li Y. (2025). Molecular epidemiology and genetic characteristics of pseudorabies virus between 2021 and 2023 in Henan Province of China. J. Vet. Sci..

[33] Song C., Ye H., Zhang X., Zhang Y., Li Y., Yao J., Gao L., Wang S., Yu Y., Shu X. (2024). Isolation and characterization of Yunnan variants of the pseudorabies virus and their pathogenicity in rats. Viruses.

[34] Chen Y.C., Wu C.T., Chen J.H., Tsai C.F., Wu C.Y., Chang P.C., Yeh W.L. (2022). Diltiazem inhibits breast cancer metastasis via mediating growth differentiation factor 15 and epithelial-mesenchymal transition. Oncogenesis.

[35] Siegel J.D., Ko C.J. (2020). Diltiazem-associated photodistributed hyperpigmentation. Yale J. Biol. Med..

[36] McAuley B.J., Schroeder J.S. (1982). The use of diltiazem hydrochloride in cardiovascular disorders. Pharmacotherapy.

[37] Dunn D.M., Munger J. (2020). Interplay between calcium and AMPK signaling in human cytomegalovirus infection. Front. Cell Infect. Microbiol..

[38] Qu Y., Wang S., Jiang H., Liao Y., Qiu X., Tan L., Song C., Nair V., Yang Z., Sun Y. (2024). Newcastle disease virus infection induces parthanatos in tumor cells via calcium waves. PLoS Pathog..

[39] Wang X., Luo J., Wen Z., Shuai L., Wang C., Zhong G., He X., Cao H., Liu R., Ge J. (2022). Diltiazem inhibits SARS-CoV-2 cell attachment and internalization and decreases the viral infection in mouse lung. PLoS Pathog..

[40] Zhu L., Huang L., Zhu Y., Ding X., Zhu G. (2017). Calcium signaling involved in bovine herpesvirus 1 replication in MDBK cells. Acta Virol..

[41] Ding L., Jiang P., Xu X., Lu W., Yang C., Li L., Zhou P., Liu S. (2021). T-type calcium channels blockers inhibit HSV-2 infection at the late stage of genome replication. Eur. J. Pharmacol..

[42] Yu T., Xu B., Bao M., Gao Y., Zhang Q., Zhang X., Liu R. (2022). Identification of potential biomarkers and pathways associated with carotid atherosclerotic plaques in type 2 diabetes mellitus: A transcriptomics study. Front. Endocrinol..

[43] He B., Sun H., Bao M., Li H., He J., Tian G., Wang B. (2023). A cross-cohort computational framework to trace tumor tissue-of-origin based on RNA sequencing. Sci. Rep..

[44] He B., Zhang Y., Zhou Z., Wang B., Liang Y., Lang J., Lin H., Bing P., Yu L., Sun D. (2020). A neural network framework for predicting the tissue-of-origin of 15 common cancer types based on RNA-seq data. Front. Bioeng. Biotechnol..

[45] Deng L., Min W., Guo S., Deng J., Wu X., Tong D., Yuan A., Yang Q. (2024). Interference of pseudorabies virus infection on functions of porcine granulosa cells via apoptosis modulated by MAPK signaling pathways. Virol. J..

[46] Xu L., Tao Q., Zhang Y., Lee F.-Q., Xu T., Deng L.-S., Jian Z.J., Zhao J., Lai S.Y., Zhou Y.C. (2024). The host cells suppress the proliferation of pseudorabies virus by regulating the PI3K/Akt/mTOR pathway. Microbiol. Spectr..

[47] Hu H., Hu Z., Zhang Y., Wan H., Yin Z., Li L., Liang X., Zhao X., Yin L., Ye G. (2022). Myricetin inhibits pseudorabies virus infection through direct inactivation and activating host antiviral defense. Front. Microbiol..

[48] Yuan X., Wang H., Zhao Z., Li C., Wang X., Liu Y., Zhou Y., Zhu Z., Zhang Z. (2025). Paeonol inhibits the replication of bovine herpesvirus type 1 in vitro through regulating the PI3K/AKT pathway. Curr. Microbiol..

